# Eutectic Processing of Semiconductor Colloidal Nanocrystals for Energy Applications

**DOI:** 10.1021/acsenergylett.6c00100

**Published:** 2026-02-24

**Authors:** Dulanjan Harankahage, William Martin, Edmund Elce, Siddhartha Thennakoon, Bhanuka Thennakoon, Maxwell Marshal Kannen, Natalia Kholmicheva, Barbra Kayira, Amelia D. Waters, Divesh Nazar, Jiamin Huang, Pavel Anzenbacher, Anton V. Malko, Mikhail Zamkov

**Affiliations:** † The Center for Photochemical Sciences, 1888 Bowling Green State University, Bowling Green, Ohio 43403, United States; ‡ Department of Physics, 1888 Bowling Green State University, Bowling Green, Ohio 43403, United States; § Department of Chemistry, 1888 Bowling Green State University, Bowling Green, Ohio 43403, United States; ∥ Department of Physics, The University of Texas at Dallas, Richardson, Texas 75080, United States; ⊥ 478201 First Solar Inc., 28101 Cedar Park Blvd, Perrysburg, Ohio 43551, United States

## Abstract

Colloidal semiconductor nanocrystals (NCs) offer a cost-effective platform for light-energy conversion in X-ray scintillators, photovoltaics, lasers, and display technologies. Yet, device-relevant NCs often require complex heterostructured compositions, where lattice imperfections compromise the efficiency and stability of photoconversion processes. Here, we show that a simple synthetic detour through a eutectic state of II–VI semiconductor NCs (e.g., CdSe, ZnSe) with halide salts (e.g., CdCl_2_, ZnCl_2_) overcomes this limitation by melting and reconstructing NC lattices into defect-free alloyed and core/shell architectures. Applied to ternary CdSeTe NCs, this process produces downconverters with record brightness and minimal line widths, delivering a 3-fold increase in film-side external quantum efficiency of commercial CdTe photovoltaic modules (First Solar Inc.). Meanwhile, eutectic processing of CdSe-based core/shell emitters yields an 8-fold enhancement in their photoluminescence stability under backlight operation, addressing the reliability bottleneck for display technologies. Together, these findings establish eutectic NC processing as a scalable route to efficient, durable photoconversion materials for energy applications.

## Graphical abstract

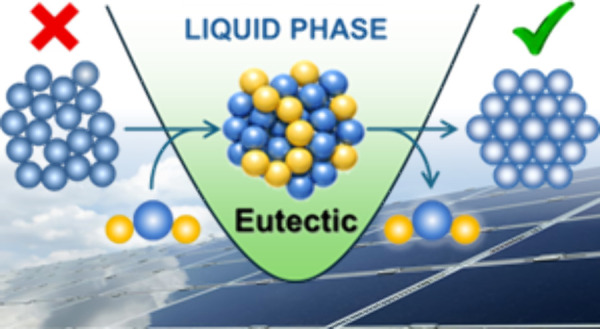

Colloidal semiconductor nanocrystals (NCs)^1^ represent a promising platform for solution-processed optoelectronics,^2–10^ with growing success in energy downconversion technologies, such as X-ray scintillators,^11^ solar concentrators,^12,13^ optically pumped lasers,^5,14^ and displays.^15,16^ These applications exploit NC properties tunable by size, composition, and heterostructure design^17–19^ - characteristics that are generally difficult to control using conventional, kinetically driven syntheses.^1,20–24^ Particularly challenging are device-relevant core/shell or alloyed systems, where lattice strain and unequal precursor reactivities hinder the intended NC growth and ultimately degrade the efficiency and stability of energy downconversion processes. Advancing these technologies, therefore, calls for a shift from kinetically limited pathways to thermodynamically guided synthesis, where NCs evolve toward stable, low-Gibbs-energy configurations.

Recent efforts to move beyond kinetic growth have indeed shown that thermodynamic pathways produce better-performing X-ray scintillators^25^ and optically pumped lasers.^26^ Such thermodynamic growth of colloidal semiconductors has been demonstrated most clearly through a liquid-like fusion of colloidal PbS and CdX (X = S, Se, Te) NCs into larger, thermodynamically faceted single crystals,^27–40^ with emission line widths approaching single-particle limits.^30,41^ Interestingly, trace halide species (e.g., CdCl_2_, PbCl_2_) have been identified as spontaneous initiators of such NC fusion (coalescence) under otherwise typical growth conditions;^28,30,40^ however, the molecular-level mechanism governing the role of halides remained unclear. An interesting parallel exists in the CdTe thin-film photovoltaics industry, where the ubiquitous CdCl_2_ annealing step (dubbed “magic step”) drives grain-boundary fusion,^42–45^ producing nearly an order-of-magnitude enhancement^46^ in polycrystalline CdTe solar cell efficiency. Only recently has evidence pointed to a plausible explanation that chloride defuses into CdTe grain boundaries and forms a localized eutectic with semiconductors, dramatically lowering the local melting point of bulk CdTe (≈1040 °C) to 400 °C in the presence of CdCl_2_,^44^ causing lattice reorganization.^42,47,48^ In the case of semiconductor colloids, melting-point depressions are even more pronounced,^49^ potentially making such a eutectic molten state accessible within the typical NC growth range of 180–320 °C. Drawing from this insight, we hypothesize that halide-induced eutectic melting could be applied to colloidal NCs toward removing defects and driving thermodynamically controlled shape evolution.

Here we show that the eutectic reconstruction of colloidal semiconductor NCs yields efficient and stable photoconversion materials for energy applications. This process is initiated by combining II–VI semiconductors (e.g., CdS, CdSe, CdTe, ZnSe) with halide salts, MX_n_ (M = Cd, Zn, In, H; X = Br, Cl, I) that, under appropriate conditions, form a transient eutectic phase. This intermediate causes melting and subsequent rearrangement of the lattice healing defects, reshaping NCs into their lowest-energy morphologies. Developed as a colloidal analogue of industrial thin-film processing,^42–46^ this route enables the growth of challenging NC compositions, including ternary alloyed NCs (Figures 1b, S1, S9d), multishell NCs (Figures 1c-d, S2, S3), and giant binary NCs (Figure 1e) with structural and optical characteristics superior to those fabricated by conventional hot-injection or heat-up methods. These improvements translate directly into device-relevant outcomes. For CdSe-based core/shell emitters, eutectic processing yields an 8-fold stability enhancement, observed in backlit display tests, which addresses a key durability bottleneck for this technology. Meanwhile, eutectic synthesis of CdTe_*x*_Se_1–*x*_ alloy NCs results in near-infrared downconverters with record brightness and minimal line widths, delivering nearly a 3-fold increase in film-side external quantum efficiency and a 0.8–1.2% absolute efficiency gain in First Solar CdTe modules.

**Figure 1:**
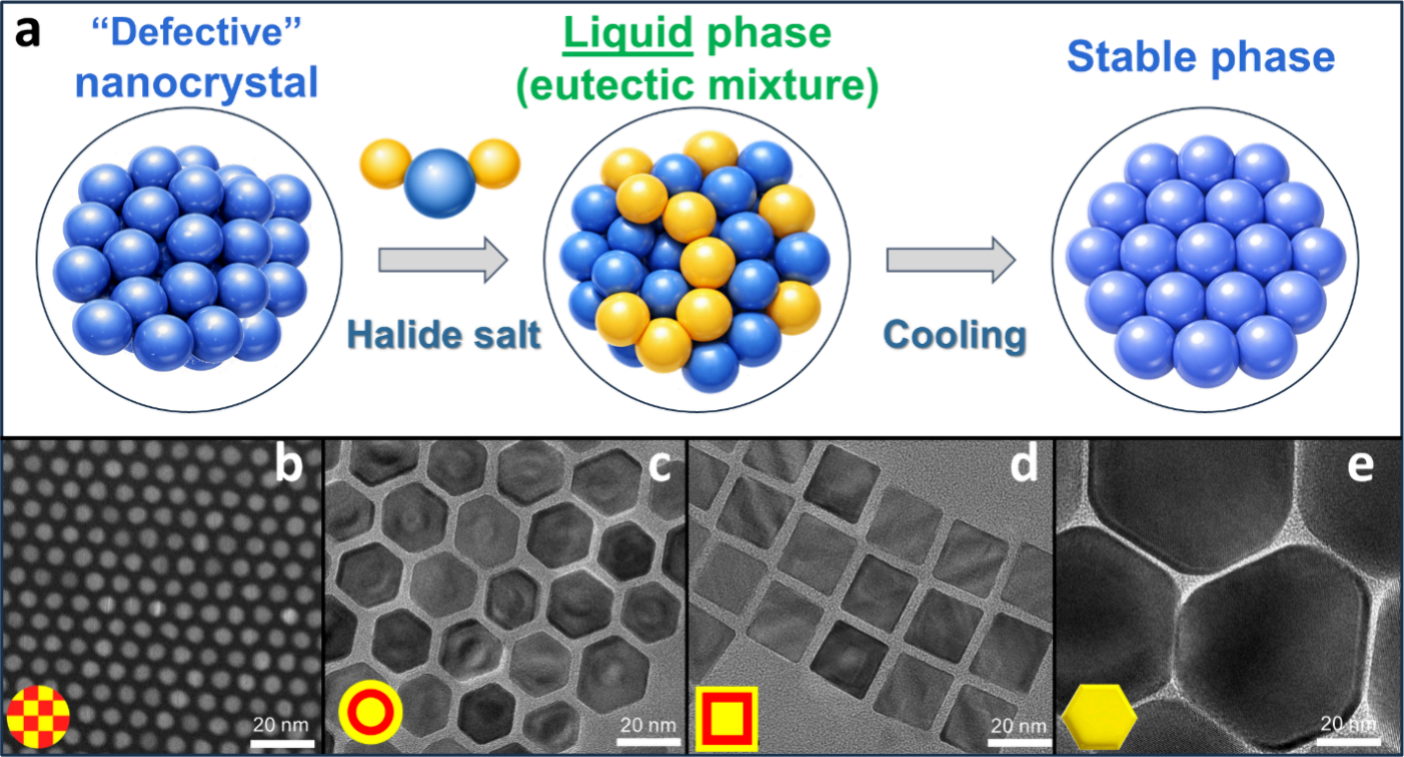
(a) Schematic illustration of the eutectic reconstruction, in which the addition of halide salts to a colloidal NC solution induces an NC-halide eutectic mixture that recrystallizes into a stable, defect-free crystalline phase. (b-e) TEM images of the representative examples of “challenging” NC morphologies synthesized via thermodynamically driven, eutectic reconstruction: (b) CdSeTe alloy NCs; (c) CdS/CdSe/CdS core/shell/shell quantum shells (QSs); (d) cubic CdS/CdSe/CdS core/shell/shell NCs; (e) giant CdS NCs.

To demonstrate NC eutectics, small-diameter CdS NCs were mixed with a CdCl_2_ salt in oleylamine (OLAM) and gradually heated to ∼320 °C while monitoring particle size. Transmission electron microscopy (TEM) images taken at successive reaction stages (Figure 2a) reveal a stepwise growth of CdS NCs from 3.5 nm (30 °C) to 4.8 nm (190 °C), 9.4 nm (260 °C), and 18.4 nm (320 °C). Notably, no evidence of Ostwald ripening^50^ (dissolution of smaller NCs in favor of larger ones) or digestive ripening^51^ (redistribution toward smaller sizes) was observed at any stage. Instead, after 3–5 min exposure of NCs to each successive reaction temperature, a single particle size was obtained (standard size deviation <7%). This suggests that the growth proceeds via direct coalescence of liquid-like NCs into a thermodynamically preferred size state determined solely by the reaction temperature (Figure 2a).

**Figure 2:**
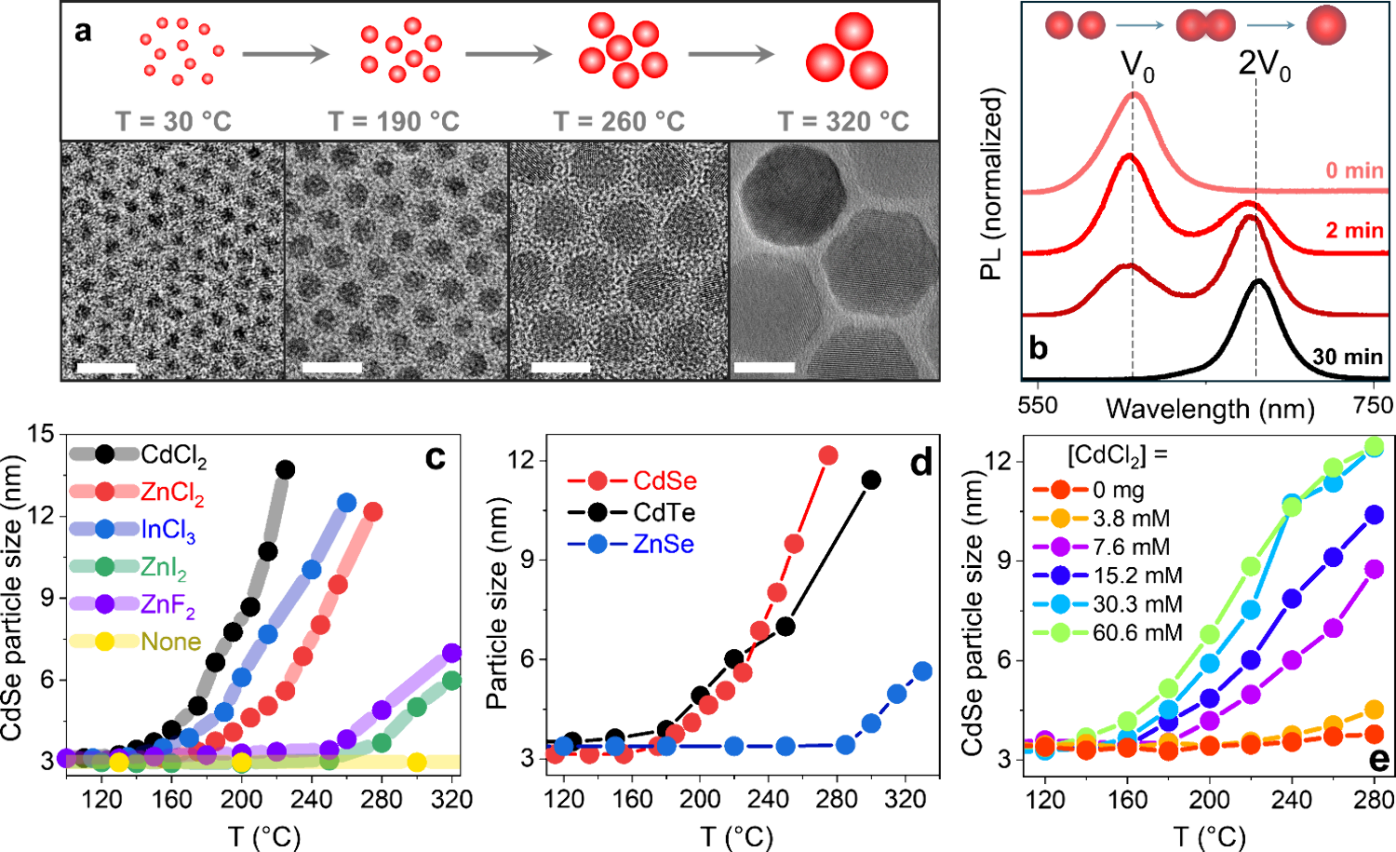
(a) Illustration of the (eutectic) coalescence mechanism, in which primary NCs fuse into larger particles with sizes determined by the reaction temperature, along with TEM images of CdS NCs taken at successive reaction temperatures, showing stepwise size increases from 3.5 nm to ≈4.8 nm, ≈9.4 nm, and ≈18.4 nm. Scale bars: 10 nm. The growth proceeds without evidence of Ostwald ripening^50^ or digestive ripening,^51^ with each temperature producing a single, uniform particle size dictated by a thermodynamically preferred state. (b) The onset of coalescence in zinc blende CdSe NCs at ≈200 °C shows gradual doubling of particle volume. (c) The effect of various halide salts on the particle size evolution during heat-up of 3 nm CdSe NCs in OLAM. In the absence of halides (yellow), no significant size change is observed. Halide addition induces temperature-dependent growth, with CdCl_2_ (gray) producing the fastest size increase, and ZnI_2_ and ZnF_2_ (green and magenta) yielding slower growth. (d) Particle size evolution during heat-up growth of ∼3 nm CdSe (red), CdTe (black), and ZnSe (blue) NCs in a mixture of ZnCl_2_ and OLAM. CdSe and CdTe exhibit similar onsets at ≈170 °C, whereas ZnSe shows a higher threshold near 280 °C. (e) Effect of the halide concentration on CdSe eutectic growth in CdCl_2_/OLAM.

A liquid-like fusion of colloidal NCs is further evident in slow-rate growth experiments in Figure 2b, performed just above the coalescence onset temperature (*T*_th_). When ∼3 nm CdSe NCs are heated in the presence of halides to about 200 °C, the original photoluminescence (PL) peak gradually vanishes, giving rise to a red-shifted feature, which corresponds to doubling of the particle volume. The presence of two discrete size populations is characteristic of coalescence, where particle fusion occurs in distinct steps rather than through continuous dissolution and reprecipitation. Throughout this process, no evidence emerges for populations corresponding to partial multiples of the original NC volume, implying that coalescence proceeds directly to the size state set by the reaction temperature. Similarly, the absence of discrete size populations corresponding to aggregates of 3–4 starting particles indicates that coalescence is not a stochastic aggregation process. At intermediate temperatures, PL spectra reveal transient bimodal profiles (Figure S4a) in which the original small-particle feature persists alongside a red-shifted band from newly coalesced structures. At the final *T* = 240 °C, the mixture was allowed to size focus for 3–5 min, leading to a narrow PL line width (Figure S4a). Together, these observations confirm that halide-induced coalescence is governed by discrete thermodynamic size minima, with temperature acting as the primary selector of NC dimensions.

The role of halides in directing growth by coalescence is further explored in Figure 2c, showing the evolution of a particle size, calculated from the evolution of NC absorption profiles (Figure S5).^52^ In the absence of halides, heating of 3 nm CdSe NCs in OLAM produces no measurable change in size, even at the highest temperatures studied (Figure 2c, yellow curve), confirming that OLAM alone does not promote coalescence. By contrast, all halide-containing reactions exhibited a pronounced temperature-dependent size increase (Figure 2c). Among the salts tested, CdCl_2_ induced the earliest onset of coalescence, driving growth well below 200 °C (gray curve), whereas ZnI_2_ and ZnF_2_ promoted slower size evolution, requiring higher temperatures (about 250 °C) to achieve comparable particle enlargement. The semiconductor material was another factor affecting the thermal threshold for coalescence. As shown in Figure 2d, CdSe and CdTe NCs exhibit nearly identical behavior in the ZnCl_2_/OLAM reaction mixture, with coalescence initiating sharply at ∼170 °C (red and black curves). In contrast, similar-size ZnSe NCs require much higher temperatures, with an onset occurring only at 280 °C (blue curve). Additionally, the halide concentration also affected the onset and the rate of growth (Figures 2e), where particle size was estimated from the spectral position of the PL in Figure S6. Notably, the higher concentrations resulted in lower onset temperatures, *T*_th_, until reaching saturation for [CdCl_2_] > 10 mM.

Having established necessary conditions for NC-halide eutectics, we have exploited this process toward improving the NC quality. Figures S4b and S4c compare CdSe growth with and without CdCl_2_ under otherwise identical conditions. In a conventional hot-injection synthesis performed at *T* = 260 °C (Figure S1b), we observe a nearly constant PL line width throughout the growth reaction (blue curve). By contrast, adding CdCl_2_ narrows the line width by 40% (red curve), consistent with size focusing and directly beneficial for energy-downconversion technologies such as display phosphors^53^ and luminescent solar concentrators.^54^ Notably, such PL narrowing occurs without long ripening periods typical of classical syntheses, suggesting that eutectic growth bypasses key kinetic bottlenecks. Similar trends were observed for several other NC/halide combinations (Figure S5).

The advantages of eutectic synthesis are also seen during the heat-up growth of CdSe NCs in Figure S4c. In the conventional precursor heat-up route (to 280 °C), the PL line width remained constant throughout the growth phase (Figure S4c, blue curve). By contrast, the addition of CdCl_2_ in an otherwise identical reaction initially produced a transient broadening of the PL spectrum, reflecting the formation of a bimodal size distribution. This was followed by line width narrowing, ultimately yielding a PL line width about 40% smaller than without CdCl_2_. Collectively, the trends observed in Figures S4a-c demonstrate that eutectic growth of binary NCs leads to improved emission characteristics compared to purely kinetic growth, irrespective of whether the synthesis is initiated via hot-injection or heat-up methods. Notably, the addition of various surfactants during the eutectic growth causes the molten NCs to recrystallize into unique shapes dictated by surface/bulk energy balance (Figure S7).

We next examine ternary alloy NCs, where even modest compositional inhomogeneity can degrade photoconversion efficiency and stability.^55–58^ In conventional syntheses, such alloys commonly develop gradients due to mismatched precursor reactivities and lattice parameters.^59^ Here, we explore a representative example of a synthetically challenging composition, CdTe_*x*_Se_1–*x*_ alloy. This ternary quantum dot (QD) is a desirable photoconversion material, which exhibits a strong optical bowing effect (Figure S9a), allowing emission tuning across the near-infrared (700–900 nm).^60,61^ Although lead- and mercury-based chalcogenides can access similar emission ranges, narrow-band mercury-based emitters (e.g., HgTe NPLs^62^ are generally not accepted for industrial solar manufacturing, while lead chalcogenides exhibit broad line widths. In contrast, CdTe_*x*_Se_1–*x*_ combines an industry-relevant composition with comparatively narrow-band emission, making it particularly well suited for light-energy conversion.

To demonstrate eutectic synthesis of CdTe_*x*_Se_1–*x*_ QDs, ∼3 nm CdSe and ∼3 nm CdTe presynthesized NCs were mixed in the presence of halide salts (Figure 3a). When heated to ∼220 °C with CdCl_2_ (∼280 °C with ZnCl_2_), the primary NCs coalesced into a single alloy phase, as indicated by the disappearance of distinct CdSe- and CdTe-like features in the PL spectra (Figure 3b). This process reproducibly yielded CdTe_*x*_Se_1–*x*_ QDs with emission line widths (∼0.1 eV) narrower than those from optimized hot-injection syntheses (>0.15 eV, see refs 63–66 and PL quantum yields of 50–88% (Figure S8b). The PL of CdTe_*x*_Se_1–*x*_ QDs was tunable from 675 to ∼900 nm (Figure S8a) by varying the initial CdTe:CdSe NC ratio with emission at 850 nm achieved for *x* = 0.78, as determined by XRD (Figures S1e, S9c) and further confirmed by large-area energy-dispersive X-ray spectroscopy (EDAX; Figure S1c) and Inductively Coupled Plasma Optical Emission Spectrometry (ICP-OES), showing the compositional variable *x* range of 0.75–0.9. TEM analysis of alloyed NCs (Figure 3f) revealed well-defined, thermodynamically shaped morphologies, while STEM EDAX mapping (Figures 3e, S1b,d) confirmed the homogeneous distribution of Te and Se. Collectively, these observations indicate that eutectic synthesis yields ternary-alloy QDs with narrow emission line widths and homogeneous elemental distribution.

**Figure 3:**
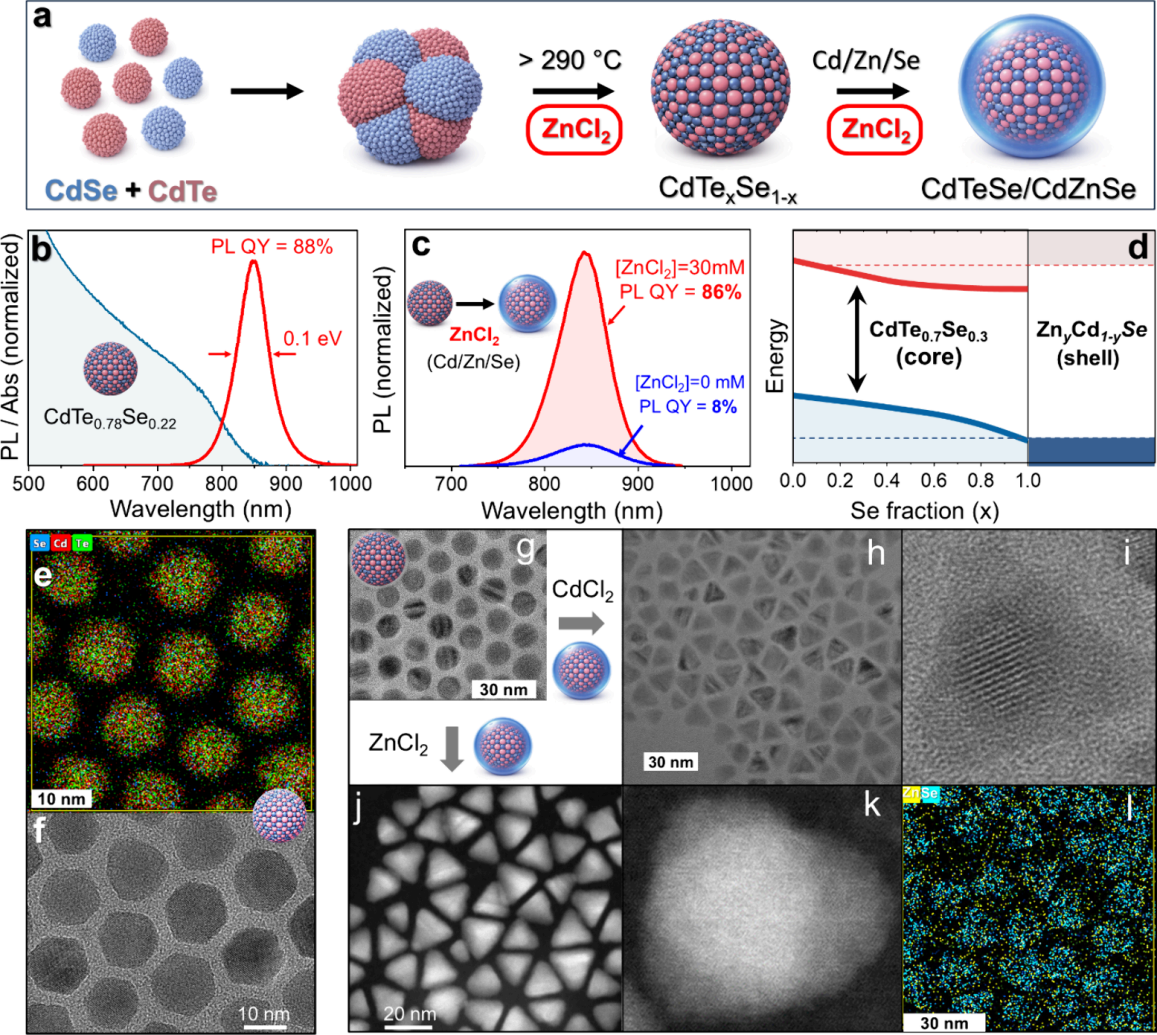
(a) Halide-assisted coalescence strategy for ternary alloy formation, in which presynthesized CdSe and CdTe NCs are reacted above the threshold temperature in a ZnCl_2_/OLAM mixture fusing into a single alloy phase. (b) Absorption and PL spectra of the alloyed CdTe_*x*_Se_1–*x*_, confirming the absence of isolated CdSe and CdTe NCs. (c) Comparison of the PL intensity and QY between CdTe_0.78_Se_0.22_/Zn_*y*_Cd_1–*y*_Se core/shell QDs grown by a standard hot-injection method (blue) and those grown in the presence of ZnCl_2_ (red). The halide-assisted route increases PL QY from ∼8% to ∼86%. (d). Schematic band edge alignment between CdTe_0.7_Se_0.3_ QDs (∼850 nm emission) and Zn_*y*_Cd_1–*y*_Se shell semiconductor for *y* ≈ 0.3–0.5. (e) Energy dispersive X-ray (EDXS) image reveals homogeneous anion distribution across the CdTe_0.78_Se_0.22_ QD alloy. (f, g) TEM images of CdTe_*x*_Se_1–*x*_ QDs exhibiting thermodynamically faceted morphologies. (h, i) TEM images of CdTe_0.78_Se_0.22_/Zn_*y*_Cd_1–*y*_Se core/shell QDs grown with CdCl_2_. (j, k) STEM characterization of CdTe_0.78_Se_0.22_/Zn_*y*_Cd_1–*y*_Se core/shell QDs grown with ZnCl_2_. (l) EDXS image depicting the elemental distribution of Zn and Se in the periphery of the CdTe_0.78_Se_0.22_/Zn_*y*_Cd_1–*y*_Se core/shell heterostructures.

The eutectic processing was next applied for depositing high-quality shells onto the lattice-mismatched cores. Such systems, featuring well-known examples like CdSe/ZnS,^3^ InP/ZnSe,^10,67^ CdSe/PbSe^68^ and CdTe/CdS core/shell QDs,^69^ often suffer from interfacial strain that leads to defects. The ternary CdTe_*x*_Se_1–*x*_ NCs described above represent a particularly challenging “model system” for shell deposition. Virtually all available wide-band gap shell semiconductors impose a substantial lattice mismatch. We have identified a Zn_*y*_Cd_1–*y*_Se composition as the closest match for a CdTe_0.78_Se_0.22_ core, still, however, exhibiting an up to 9% strain depending on composition variable *y* (Figure 3d). Conventional shell growth in this case is limited by the two competing processes: at high temperatures required for epitaxy, selenium can fully exchange with tellurium in the core, erasing the intended composition, whereas lower-temperature growth yields defective shells. The latter scenario is evident in Figure 3c (blue curve), where a standard hot-injection synthesis results in a PL QY of only ∼8%.

In contrast, the introduction of a halide salt (ZnCl_2_) into the same reaction medium fundamentally changes the growth pathway. Properly tuned eutectic conditions led to a reduction in the local melting point at the core/shell interface without affecting the entire NC, leading to strain-relieved shell deposition (see Section S1). In this work, such conditions were realized at *T* ≈ 225 °C, allowing CdTe_0.78_Se_0.22_ cores to be successfully overcoated with Zn_*y*_Cd_1–*y*_Se, as reflected by a dramatic increase in PL QY to ∼86% (Figure 3c, red curve; Figure S10). Similarly, microscopy images of core/shell QDs grown in the presence of CdCl_2_ (Figures 3h, 3i) or ZnCl_2_ (Figures 3j-3l) reveal well-faceted, thermodynamically evolved shells with high-symmetry morphologies, extending the scope of halide-assisted growth to strain-tolerant core/shell nanostructures.

Eutectic synthesis of CdTe_0.78_Se_0.22_/Zn_*y*_Cd_1–*y*_Se NCs yields an important material for spectral shaping, which is relevant to the CdTe photovoltaics industry. The film-side external quantum efficiency (FS-QE) curve of the baseline commercial CdTe PV modules from First Solar Inc. exhibits a single, narrow peak centered at approximately 850 nm, with a generally low response across the visible spectrum (Figure 4c, blue curve). Introducing CdTe_0.78_Se_0.22_/Zn_*y*_Cd_1–*y*_Se NCs that emit around 850 nm broadens the FS-QE and produces pronounced visible-range enhancement (Figure 4c, yellow curve) by downconverting high-energy photons into NIR that the device harvests efficiently. We demonstrated that the application of CdTe_0.78_Se_0.22_/Zn_*y*_Cd_1–*y*_Se NCs to the back side of the solar cell (Figures 4b, S11) enables the film-side short-circuit current to rise from 3.3 mA cm^–2^ to 9 mA cm^–2^ (Figure 4d). This gain corresponds to a 0.8–1.2% absolute increase in the power conversion efficiency of the First Solar CdTe module (see eq S1). In effect, CdTe_0.78_Se_0.22_/Zn_*y*_Cd_1–*y*_Se NCs acted as spectrally conformal “photon funnels,” which broadened the FS-QE response and increased the film-side photocurrent.

**Figure 4:**
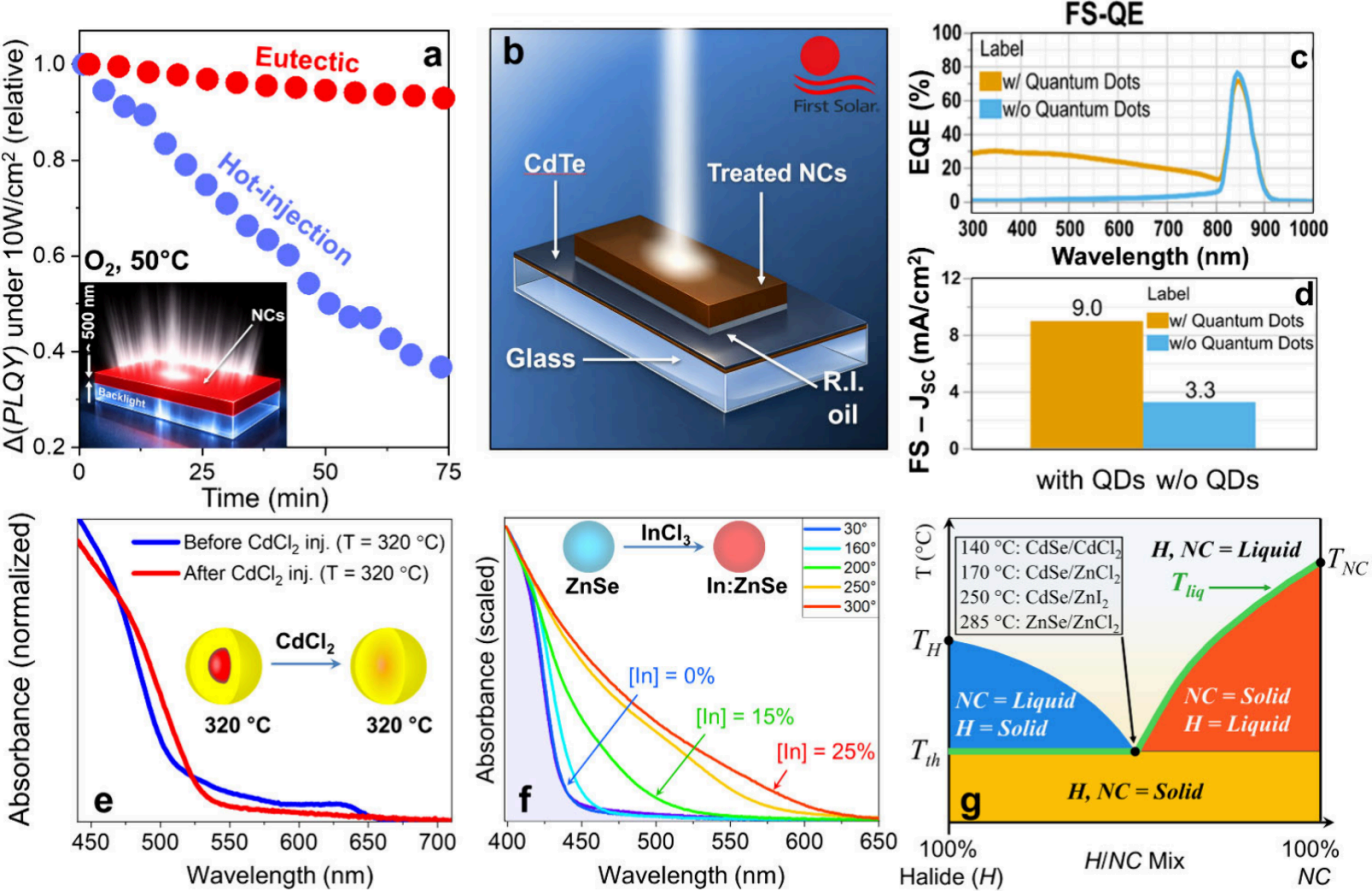
(a) Time evolution of the PLQY for eutectic-processed (red) and untreated (blue) NC films (∼500 nm thick) under 50 °C and 10 W cm^–2^ laser irradiation. Films were unencapsulated (air exposure). Initial PLQY was 63% (Edinburgh FS5 with integrating sphere). (b) Schematic of the experimental setup to acquire film side quantum efficiency (FS-EQ) using a QD solution. (c) Film side quantum efficiency of a commercially available CdTe solar cell with and without QDs. (d) Film side short circuit current of a CdTe solar cell with and without QDs. (e). Estimating the halide ion diffusion in giant CdSe/CdS core/shell NCs (2.8 nm core, 3.5 nm shell). Heating NCs to 320 °C in OLAM alone (blue curve) preserves distinct CdSe and CdS absorption features. Upon the injection of CdCl_2_ at this temperature (red curve), the CdSe feature vanishes within 1 min, indicative of full-particle liquefaction. (f). Cation diffusion during eutectic growth is analyzed using ZnSe NCs’ reaction with InCl_3_ in OLAM. Progressive red-shifting of the ZnSe absorption feature with increasing temperature reveals a partial cation exchange, reaching ∼25% Zn-to-In substitution at 320 °C. (g). Qualitative eutectic phase diagram for halide (H)–nanocrystal (NC) mixtures, with the halide fraction on the *x*-axis and reaction temperature on the *y*-axis. Four regimes are identified: (I) below the eutectic threshold temperature (*T*_th_), both phases remain solid (yellow); (II) *T*_th_ < *T* < *T* ^H^ NCs partially liquefy while halides remain solid (blue); (III) *T*_th_ < *T* < *T* ^NC^ halides melt while NCs remain crystalline (red); (IV) *T* > max (*T* ^H^, *T* ^NC^) both phases are liquid, enabling complete intermixing (light gray).

Eutectic processing was also instrumental in enhancing the stability of core/shell NC architectures, which is crucial for display technologies.^54^ We have compared the PL deterioration of CdS/CdSe/CdS quantum shells (Figure 1c) grown from CdS cores prepared either by hot-injection (kinetic growth) or by halide-assisted eutectic growth (thermodynamic mode). Films of each sample (∼500 nm thickness) were cast on a standard backlight test kit, placed in an integrating sphere, and irradiated from the back with a 10 W cm^–2^ UV LED in air. After ∼1 h of exposure (Figure 4a), the kinetically grown samples showed a typical PL retention of ∼30% (i.e., a 70% drop, blue circles), consistent with conventional CdSe/CdS core–shell NC behavior.^70^ By contrast, the thermodynamically grown QSs retained ∼96% of their initial PL (a ∼4% drop, red circles), indicating that the eutectic route yields shell interfaces that are resilient to photothermal stress in an unencapsulated configuration.

An improved quality of colloidal NCs under eutectic growth can be rationalized by considering the halide-NC thermodynamic environment.^71^ The chemistry of halide-mediated treatments (e.g., CdCl_2_) in the CdTe PV industry is often summarized as^72,73^

$$\mathrm{CdTe}\;\left(\mathrm{solid}\right)+{\mathrm{CdCl}}_{2}\to \mathrm{Cd}\;\left(\mathrm{liquid}\right)+\mathrm{Te}\;\left(\mathrm{liquid}\right)+{\mathrm{Cl}}_{2}\;\left(\mathrm{liquid}\right)\to \mathrm{CdTe}\;\left(\mathrm{solid}\right)+{\mathrm{CdCl}}_{2}$$

This reaction framework captures the formation of a transient eutectic phase that facilitates mass transport, recrystallization to an improved CdTe matrix, and defect healing.^74^ However, in colloidal NCs, the relevant thermodynamics and kinetics are altered by their nanoscale nature, associated with large surface-to-volume ratios and the presence of organic ligands. To develop a mechanistic model of halide-induced liquification for colloidal systems, two fundamental questions must be addressed: (i) Do halide anions diffuse into the NC lattice? and (ii) Do the cations from halide salts diffuse into the NC? Both are prerequisites for forming a true eutectic mixture within the particle rather than solely at its surface. We first estimated the halide diffusion depth using giant CdSe/CdS core/shell NCs as a model system. According to Figure 4e, in the absence of halides, heating these NCs in OLAM to 320 °C preserved the core/shell structure, as evidenced by the absorption spectrum (blue curve), showing a distinct low-energy shoulder at 650 nm (CdSe core) and a higher-energy feature at ∼500 nm from the CdS shell. Upon the injection of CdCl_2_ at this temperature, the absorption profile of the reaction product changed significantly in seconds (Figure 4e, red curve). The CdSe and CdS features vanish, giving rise to a smooth intermediate-energy band characteristic of a homogeneous CdSeS alloy. This abrupt spectral change implies that halide addition liquefies not only the surface layer but also the entire NC, erasing the core/shell boundaries (at 320 °C). To evaluate the cation diffusion process, we monitored the transformation of ZnSe NCs upon heating with InCl_3_ in OLAM. As shown in Figure 4f, this combination led to a measurable cation exchange, evidenced by a progressive red-shift in the ZnSe/InCl_3_ absorption spectrum far beyond its corresponding bulk value. Using literature-reported correlations between the spectral position and composition of In-doped ZnSe,^75^ we estimate that at 320 °C up to ∼25% of Zn sites were replaced by In. A similar trend was observed when ZnSe was reacted with CdCl_2_, in which case cation exchange proceeded to completion, yielding fully Cd-substituted particles (Figure S12).

Taken together, these findings show that both halide anions and their corresponding cations can permeate the entire NC lattice under eutectic conditions, which is essential for whole-particle liquefaction. With this in mind, we summarize the thermodynamic picture using a schematic halide-NC eutectic phase diagram in Figure 4g,^76^ where the *x*-axis represents the halide/NC composition ratio and the *y*-axis denotes the reaction solvent temperature. The exact phase boundaries depend on the NC size, lattice structure, and ligand environment and are therefore challenging to predict. However, in practice, four qualitative regimes can be distinguished. Below the eutectic threshold temperature (*T*_th_), both components remain solid. Increasing the temperature into the *T*_th_ *< T < T*_NC_ range and using a low concentration of halide mixtures (Figure 4g, red region) creates a regime where halides are molten while NCs remain crystalline, enabling halide-mediated surface wetting and enhanced diffusion of both cations and anions along the NC surface. Experimental evidence of this regime comes from the improved crystallinity of the Zn_*y*_Cd_1–*y*_Se shell, grown over a CdTe_0.78_Se_0.22_ core. Notably, a higher halide content shifts the system into a lower NC melting point (Figure 4g, green curve), lowering the onset of coalescence (*T*_liq_) until the saturation point (also the eutectic point) is reached. This behavior is consistent with the trend observed in Figure 2e. Finally, in the highest temperature regime, *T* > max­(*T*_H_, *T*_NC_), both phases are liquid, facilitating complete intermixing and full particle liquefaction. The threshold temperature for this transition was determined experimentally for selected combinations of NCs and halides (insert). We also note that the *T* > max­(*T*_H_, *T*_NC_) regime is strongly dependent on the interfacial energy between the solid (nanoparticle) and matrix (solvent) phases and can lead to either full nanoparticle melting or melting of surface layers, as explained in SI Section 1 (ref 77.).

In conclusion, we demonstrate that the eutectic reconstruction of colloidal semiconductor nanocrystals yields efficient and stable photoconversion materials for energy applications. Developed as a colloidal analogue of industrial thin-film processing, this route produces binary and ternary-alloy NCs with structural and optical quality beyond conventional hot-injection or heat-up methods and enables high-quality shells on lattice-mismatched cores with narrow PL line widths and improved PL quantum yields. This eutectic processing “reset” directly improves photoconversion performance in device technologies: CdSeTe downconverters produced by eutectic synthesis deliver brighter, spectrally narrower emission and yield 0.8–1.2% absolute efficiency gain in commercial CdTe photovoltaic modules, while CdSe-based core/shell emitters show an 8-fold improvement in photoluminescence stability under backlight operation. Taken together, these results establish eutectic growth as a scalable route to next-generation NC optoelectronics.

## Supplementary Material



## Notes

Methods Section. Additional TEM images, PL/absorption spectra, rate equations and model calculations. (PDF)
