# Supplementary material for: Eutectic Processing of Semiconductor Colloidal Nanocrystals for Energy Applications
Source: ACS Energy Lett. 2026 Feb 24;11(3):2943–51. doi: 10.1021/acsenergylett.6c00100 (PMC12993914; doi:10.1021/acsenergylett.6c00100)
Supplement: Supplementary file 1 [file nz6c00100_si_001.pdf]

Supporting Information for:

Eutectic Processing of Semiconductor Colloidal Nanocrystals for Energy Applications.

Dulanjan Harankahage[1,2], William Martin[5], Edmund Elce[5], Siddhartha Thennakoon[1,2], Bhanuka Thennakoon[1,2], Maxwell Marshal Kannen[2], Natalia Kholmicheva[5], Barbra Kayira[2], Amelia D. Waters[1,2], Divesh Nazar[1,2], Jiamin Huang[1,2], Pavel Anzenbacher[1,3], Anton V. Malko[4], Mikhail Zamkov[1,2,].*

The Center for Photochemical Sciences, Bowling Green State University, Bowling Green, Ohio 43403, USA.[1]

Department of Physics, Bowling Green State University, Bowling Green, Ohio 43403, USA.[2]

Department of Chemistry, Bowling Green State University, Bowling Green, Ohio 43403, USA.[3]

Department of Physics, The University of Texas at Dallas, Richardson, Texas, 75080, USA.[4]

First Solar Inc., 28101 Cedar Park Blvd, Perrysburg, Ohio 43551, USA.[5]

Corresponding author: zamkovm@bgsu.edu; Tel: 419-372-0264; Fax: 419-372-9938

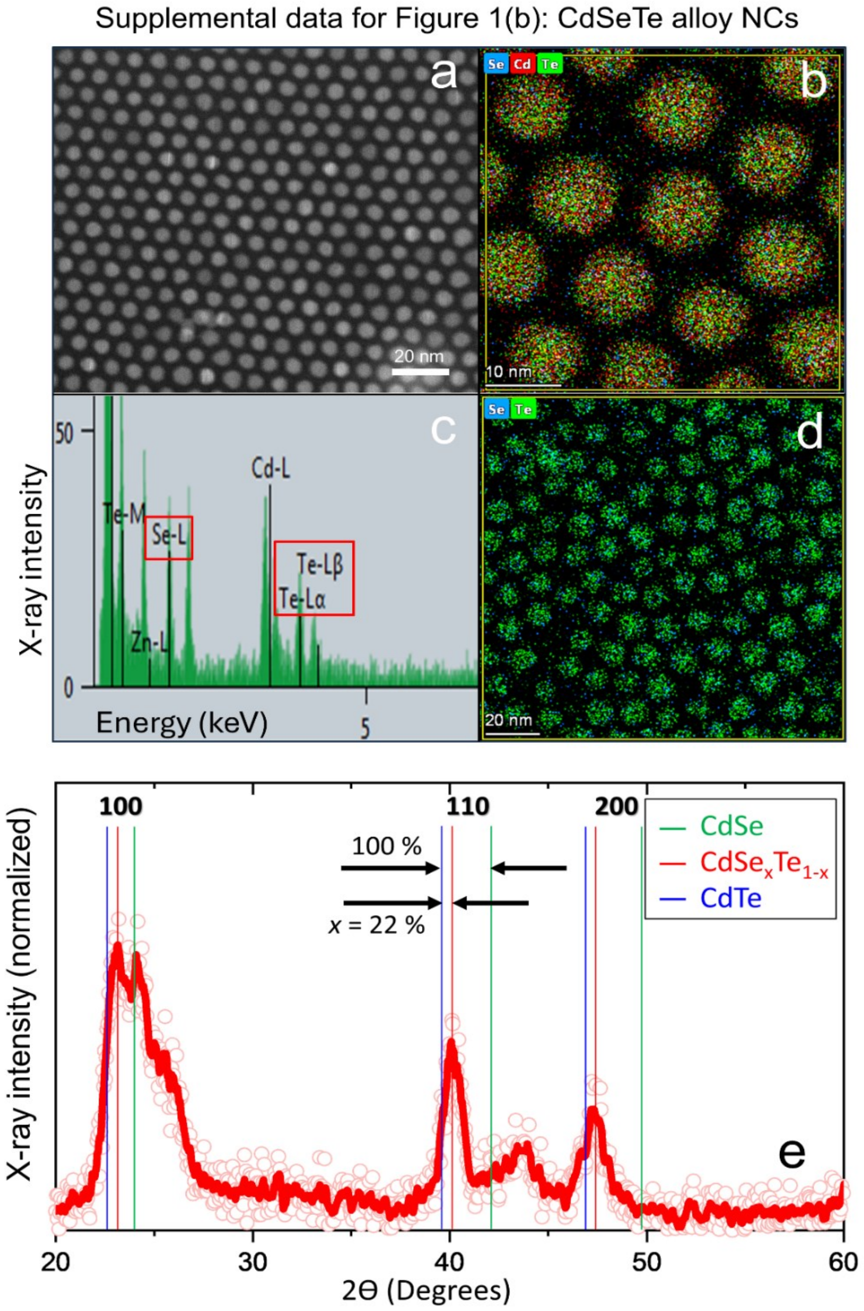

Figure S1. *Supplemental information for Figure 1b (main text)*. (a) Representative TEM images of alloyed $CdTe_{1-x}Se_x$ quantum dots (QDs). (b) HAADF-STEM (high-angle annular dark-field scanning transmission electron microscopy) image of $CdTe_{1-x}Se_x$ QDs with corresponding EDXS (energy-dispersive X-ray spectroscopy) mapping, showing a homogeneous anion distribution across individual particles. (c) EDXS elemental mapping acquired from a film of $CdTe_{1-x}Se_x$ QDs, consistent with an anion ratio of Se:Te ≈ 1:4. (d) HAADF-STEM image of $CdTe_{1-x}Se_x$ QDs with Se and Te EDXS maps, further confirming uniform alloying within individual QDs. (e) XRD spectrum of $CdTe_{1-x}Se_x$ QD powders, showing a zinc blende phase with lattice parameters between those of CdTe and CdSe; the linear fit to the peak positions is consistent with x ≈ 0.22.

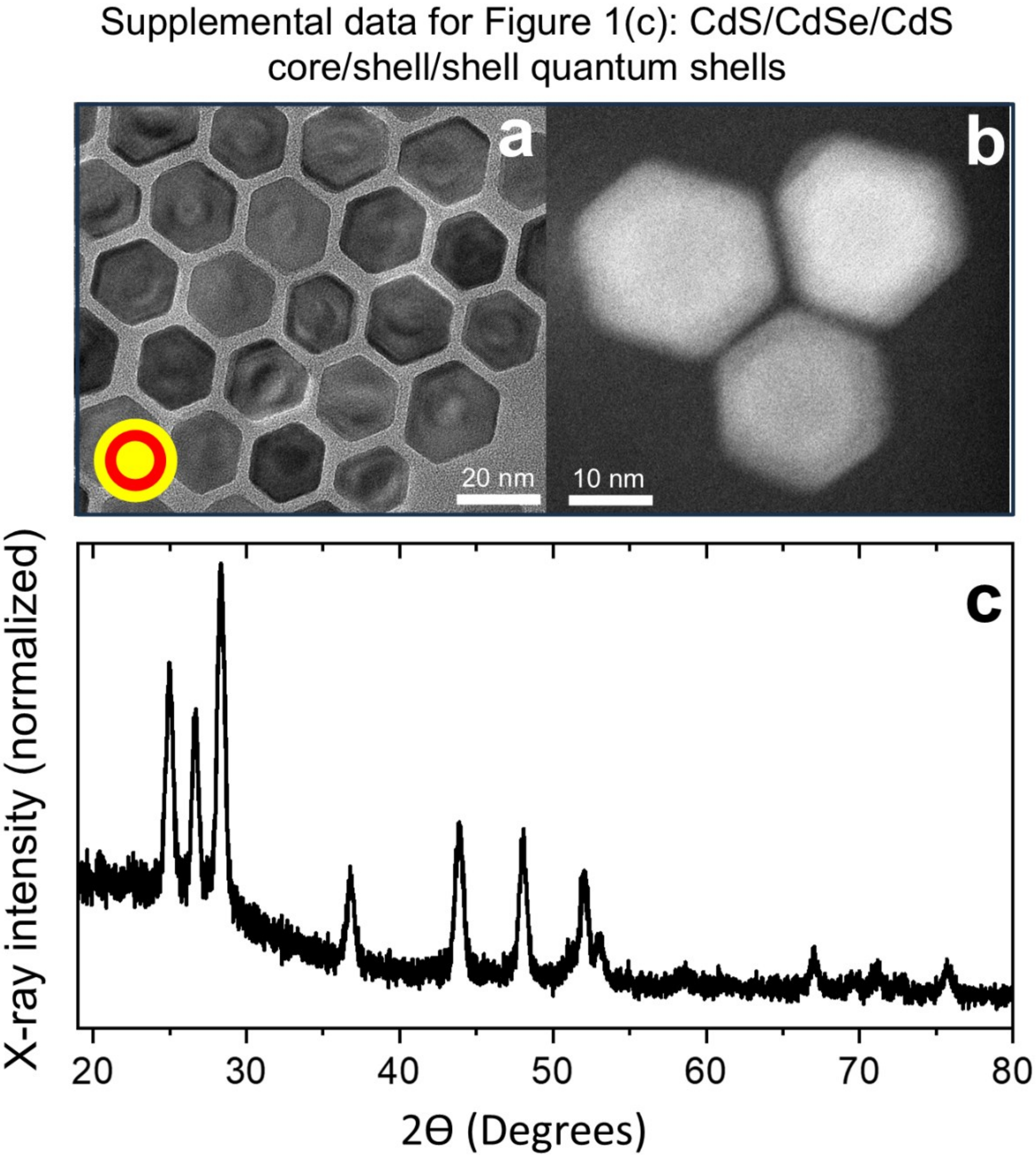

Figure S2. *Supplemental information for Figure 1c (main text)*. (a) Representative TEM images of CdS/CdSe/CdS core/shell/shell nanocrystals with CdS domains synthesized under eutectic growth conditions. (b) HAADF-STEM image of CdS/CdSe/CdS core/shell/shell nanocrystals. (c) XRD spectrum of CdS/CdSe/CdS nanocrystal powders, showing a wurtzite phase with Bragg peaks nearly matching those of bulk CdS.

Supplemental data for Figure 1(d): cubic CdS/CdSe/CdS core/shell/shell NCs.

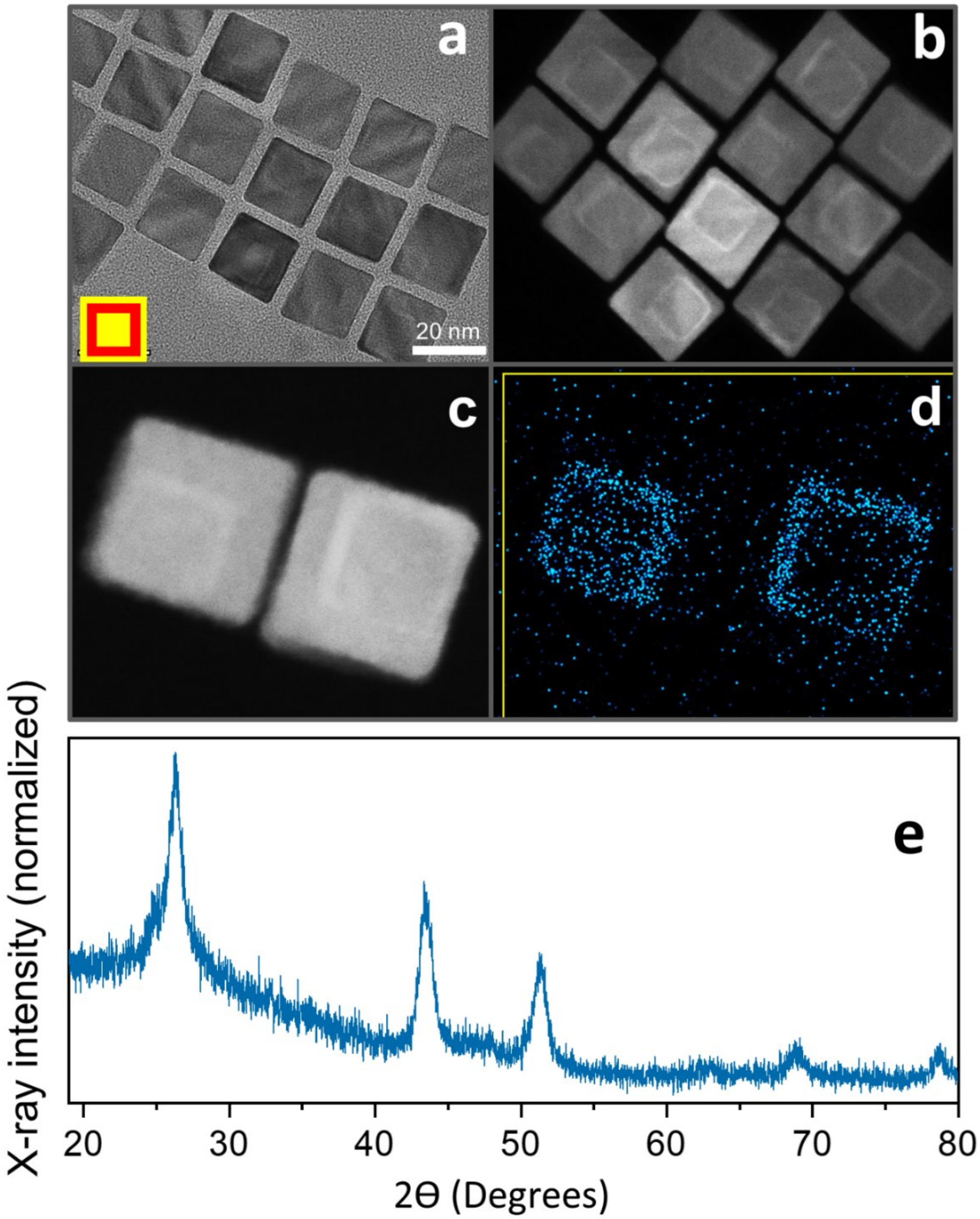

Figure S3. *Supplemental information for Figure 1d (main text)*. (a) Representative TEM images of CdS/CdSe/CdS core/shell/shell quantum cubes, with CdS domains synthesized under eutectic growth conditions. (b,c) HAADF-STEM images of CdS/CdSe/CdS quantum cubes with showing the location of the CdSe quantum well through contrast. (d) EDXS HAADF-STEM image of CdS/CdSe/CdS quantum cubes in (c), showing the location of the CdSe quantum well. (e) XRD spectrum of CdS/CdSe/CdS quantum cubes, revealing a zinc blende phase with Bragg peaks nearly matching those of bulk ZB CdS.

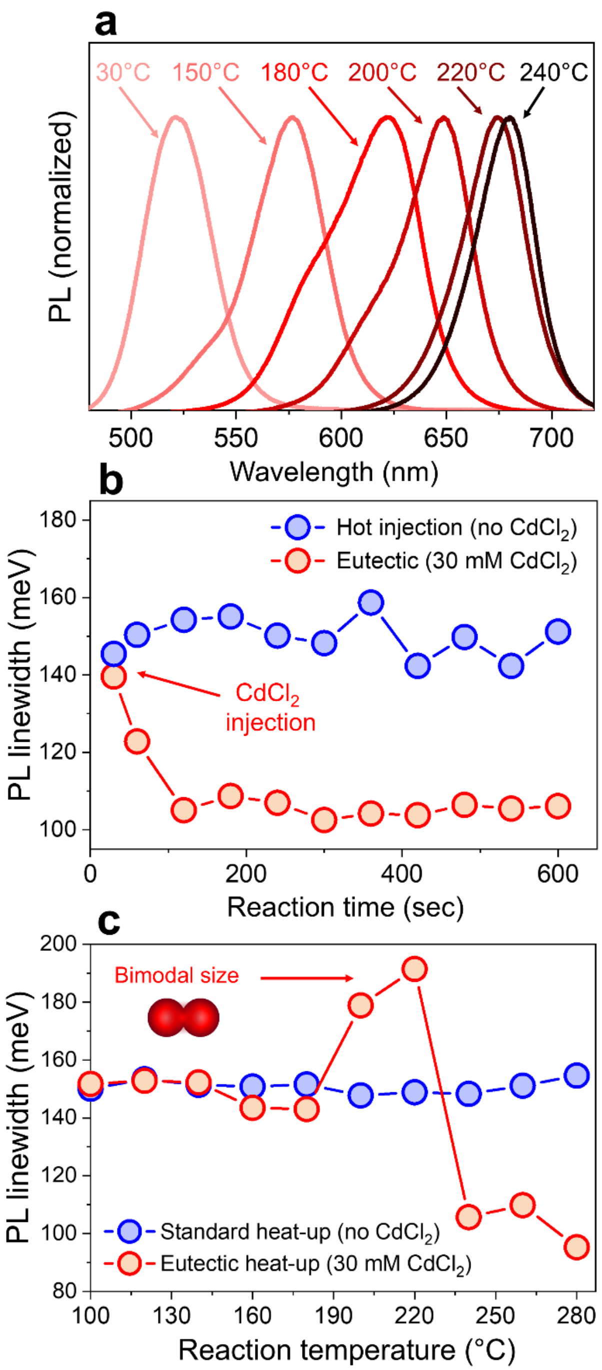

Figure S4. (a) PL spectra corresponding to the coalescence-growth of 2.6-nm CdSe NCs, recorded at intermediate temperatures (150–240°C). The trend reveals bimodal size distributions during early-stage coalescence (< 3 min after temperature increase), with a blue-shifted peak from unreacted small particles and a red-shifted peak from fused particles. (b). Evolution of the PL linewidth for CdSe NCs grown via a hot-injection route (T = 260°C). Conventional synthesis (blue curve) is compared to a eutectic growth (red curve) where $CdCl_2$ is introduced immediately after injecting the starting CdSe seed material, leading to a ~ 40% reduction in the linewidth. (c) PL linewidth evolution during a heat-up of CdSe NCs in 100% OLAM (T = 100 – 280 °C). In the absence of halides (blue curve), the linewidth remains nearly constant

throughout growth. With $CdCl_2$ present (red curve), the linewidth initially broadens due to a transient bimodal size distribution followed by progressive narrowing to about ~ 65% of the starting value.

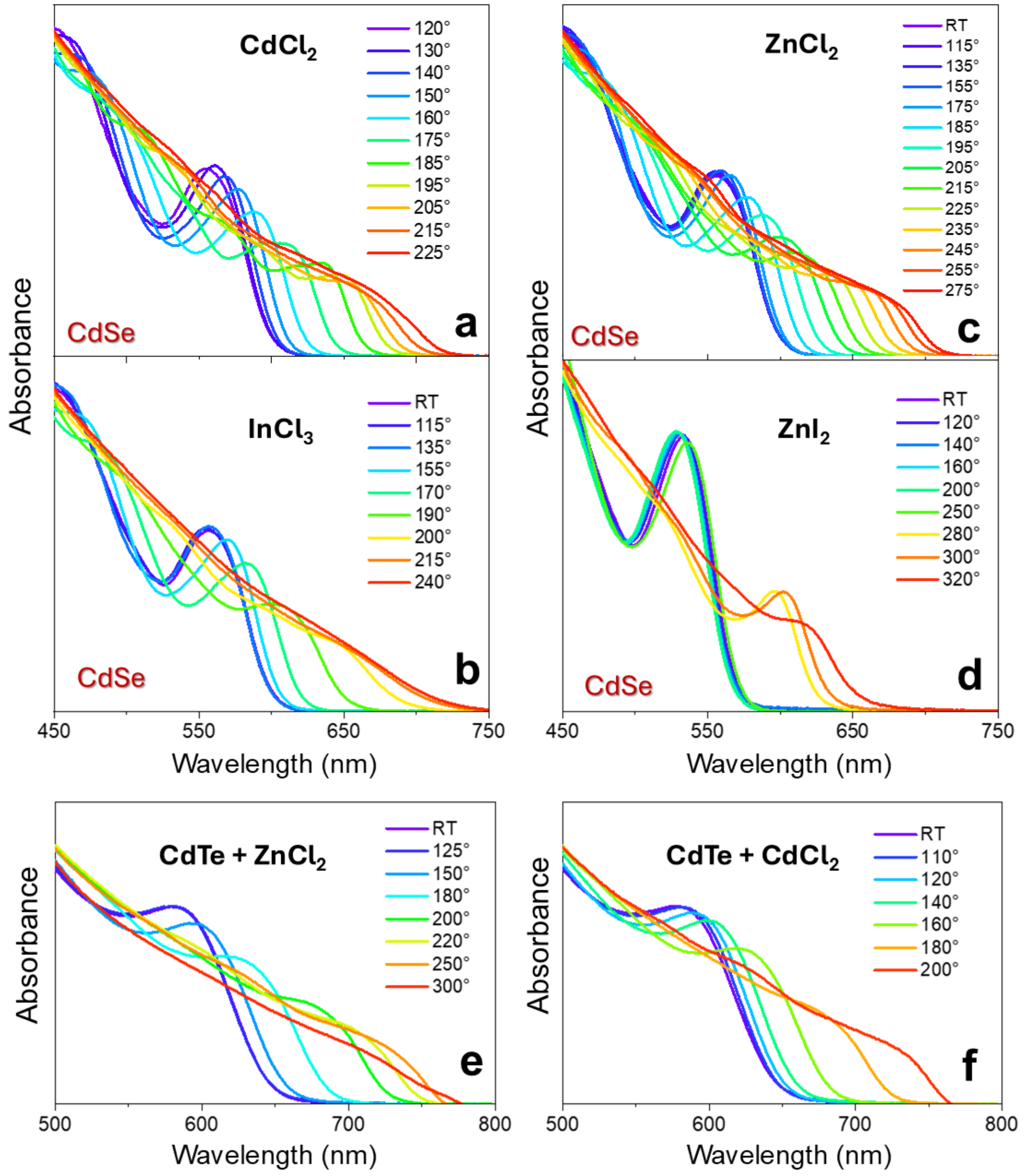

CdTe: $D = (9.8127 \times 10^{-7})\lambda^3 - (1.7147 \times 10^{-3})\lambda^2 + (1.0064)\lambda - (194.84$

CdSe: $D = (1.6122 \times 10^{-9})\lambda^4 - (2.6575 \times 10^{-6})\lambda^3 + (1.6242 \times 10^{-3})\lambda^2 - (0.4277)\lambda + (41.57)$

Figure S5. Temperature-dependent absorption evolution of colloidal NCs under halide-assisted heat-up treatment. Absorption spectra are shown as a color-graded series, where cool colors correspond to the earliest spectra and warm colors to the latest spectra in each panel. (a) CdSe NCs in the presence of $CdCl_2$. (b) CdSe NCs in the presence of $ZnCl_2$. (c) CdSe NCs in the presence of $InCl_3$. (d) CdSe NCs in the

presence of ZnI_2. (e) CdTe NCs in the presence of $ZnCl_2$. (f) CdTe NCs in the presence of $CdCl_2$. The color progression in each panel captures the temporal/thermal evolution of excitonic features and continuum absorption under the indicated halide additive. Bottom line: Particle size calculation using empirical equations from Ref (Chem. Mater. 15 2854-2860)

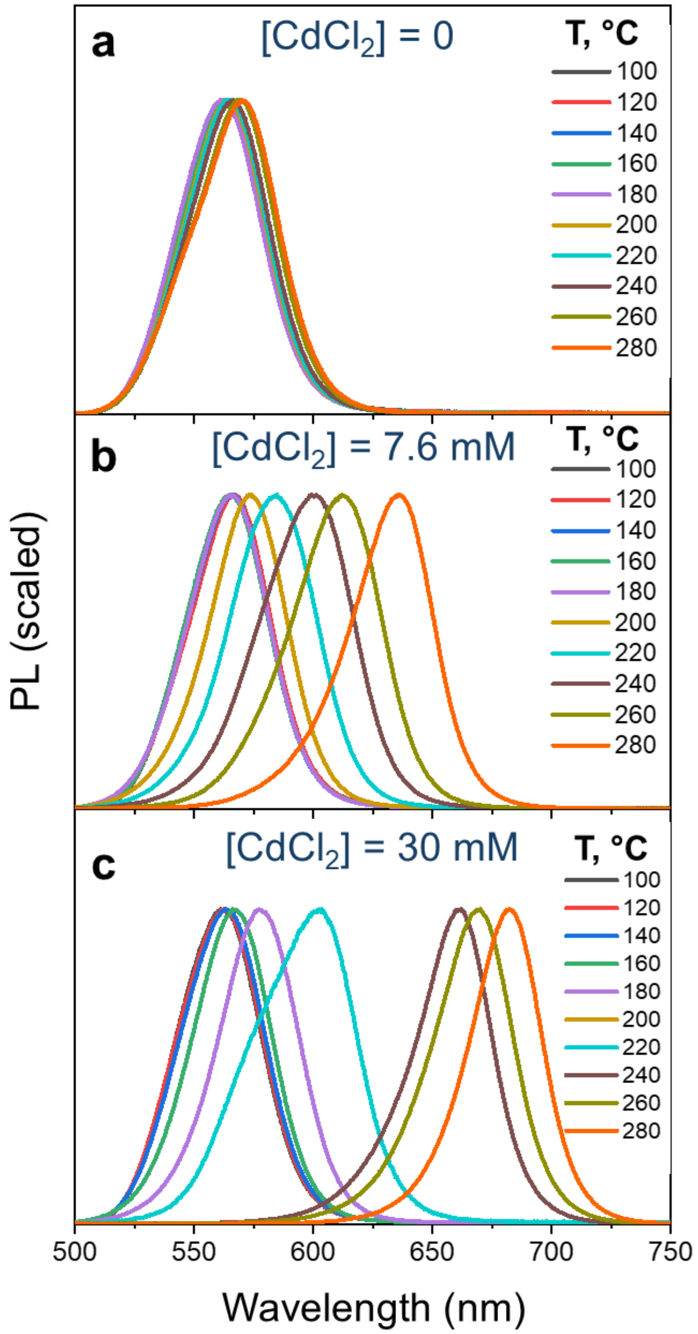

Figure S6. Comparison of the PL intensity evolution during heat up treatment of CdSe NCs in the presence of different concentration of $CdCl_2$. Panels compare CdSe NCs treated with (a) 0 M $CdCl_2$, (b) 7.6 mM

$CdCl_2$, and (c) 30 mM $CdCl_2$. Traces are color-coded to indicate progression from the start to the end of heat-up cycle.

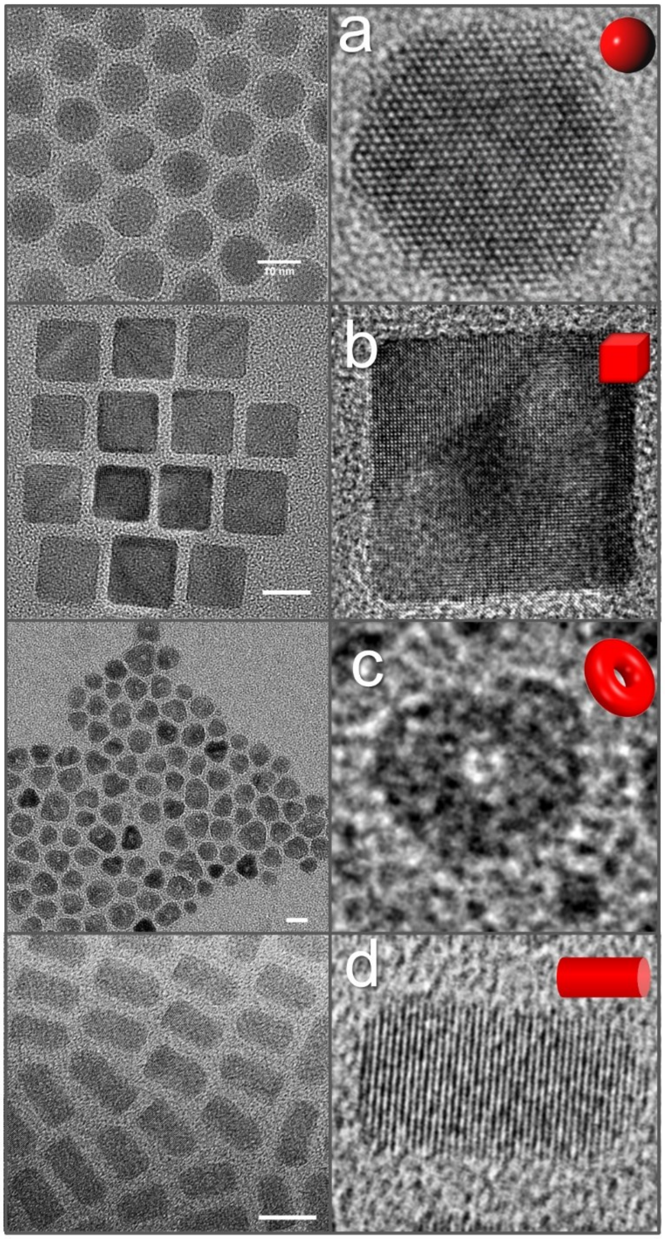

Figure S7. Shape-selective synthesis of CdS NCs by halide-assisted aggregative growth. ~3-nm zinc-blende CdS seeds were induced to coalesce under different ligand/halide environments; the coordinating surfactant dictates facet stabilization during oriented attachment, yielding four distinct morphologies at comparable precursor loadings. (a) Spheres obtained in OLAM with $CdCl_2$ at 260 °C - quasi-isotropic growth consistent with uniform surface passivation. (b) Cubes produced in OA with an acyl chloride additive ($C_{17}H_{35}COCl_{(2)}$) at 300 °C. (c) Rods grown in OLAM/$CdCl_2$ at 200 °C. The reaction proceeds to completion over 24 h, yielding anisotropic attachment and elongation along

a preferred axis. (d) Donut-like (torus) particles synthesized in sodium oleate (NaOA) at 245 °C. All panels: representative TEM images; scale bars, 20 nm. Abbreviations: OLAM = oleylamine; OA = oleic acid; NaOA = sodium oleate.

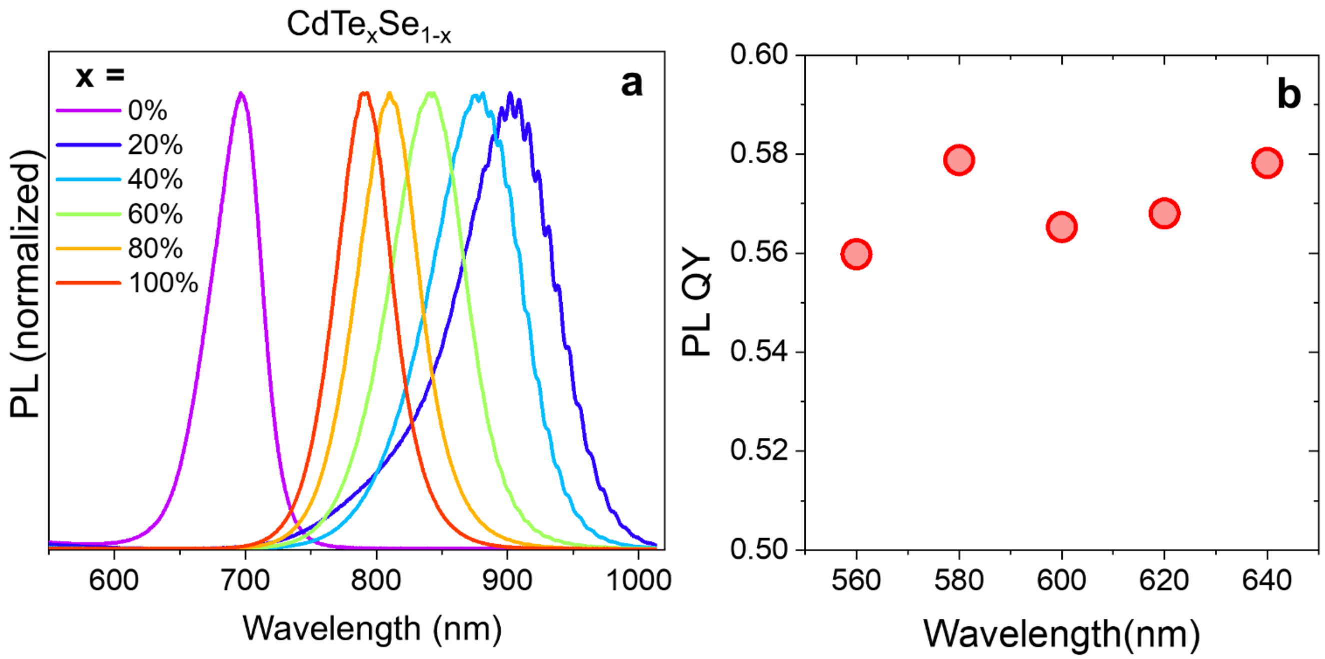

Figure S8. (a) Room-temperature PL spectra (intensity normalized) for CdTe$_x$Se$_{1-x}$ nanocrystals grown to ~ 6-7 nm in diameter, with composition spanning x = 0 → 1 (0%, 20%, 40%, 60%, 80%, 100% Te). (b) Photoluminescence quantum yield (PL QY) of CdTe$_{0.8}$Se$_{0.2}$ nanocrystals plotted versus excitation wavelength.

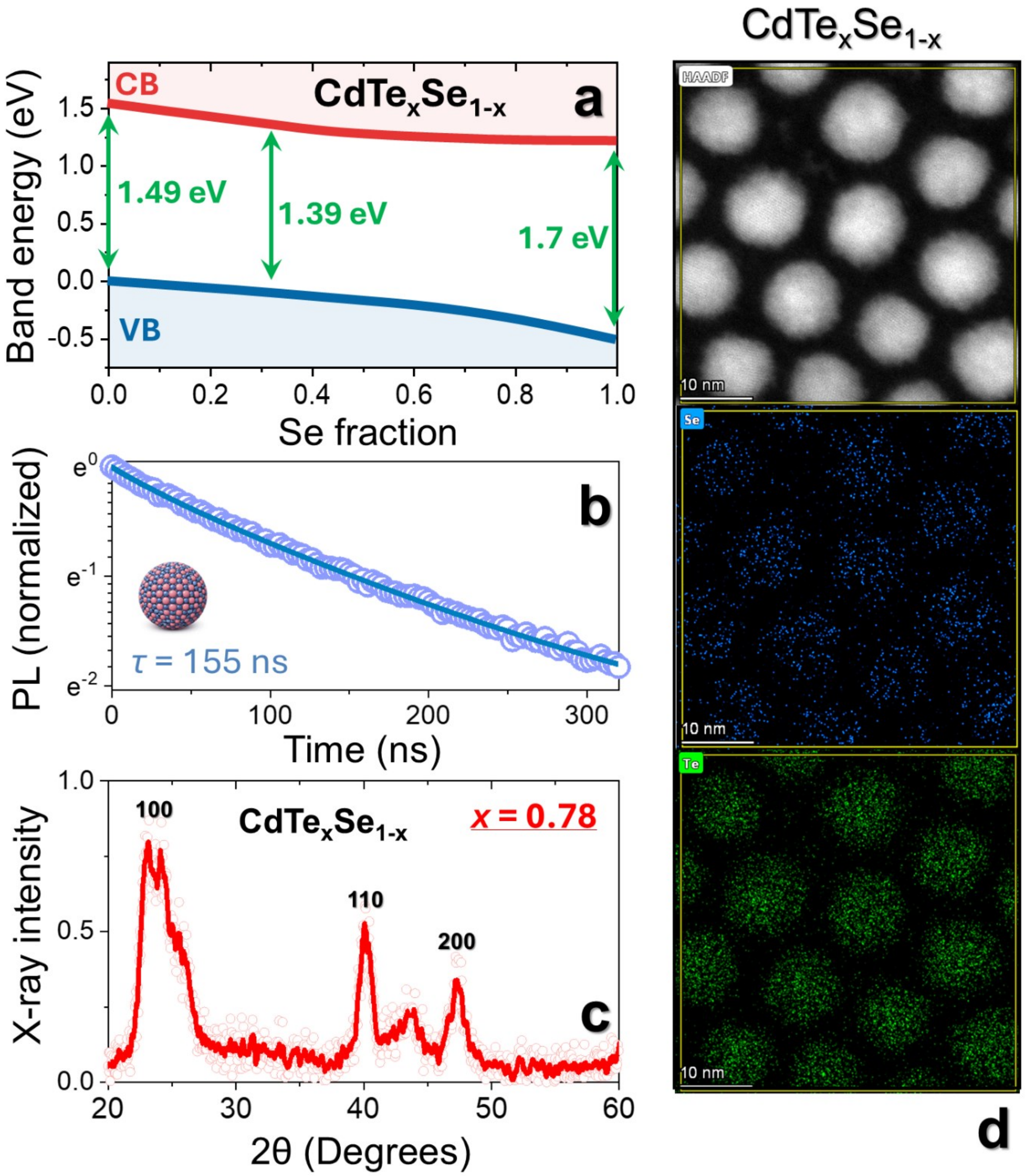

Figure S9. (a) Optical bowing in $CdTe_xSe_{1-x}$ QDs enables emission tuning from 675 nm to ~900 nm by adjusting Te:Se ratio and/or final particle size. (b) PL intensity decay of $CdTe_xSe_{1-x}$ QDs. (c) X-ray diffraction pattern of a $CdTe_xSe_{1-x}$ QD sample emitting at 850 nm with Bragg peak positions indexed to a cubic lattice with x = 0.78. (d) HAADF-STEM image of $CdTe_xSe_{1-x}$ QDs with corresponding EDXS mapping, showing a homogeneous anion distribution across individual particles.

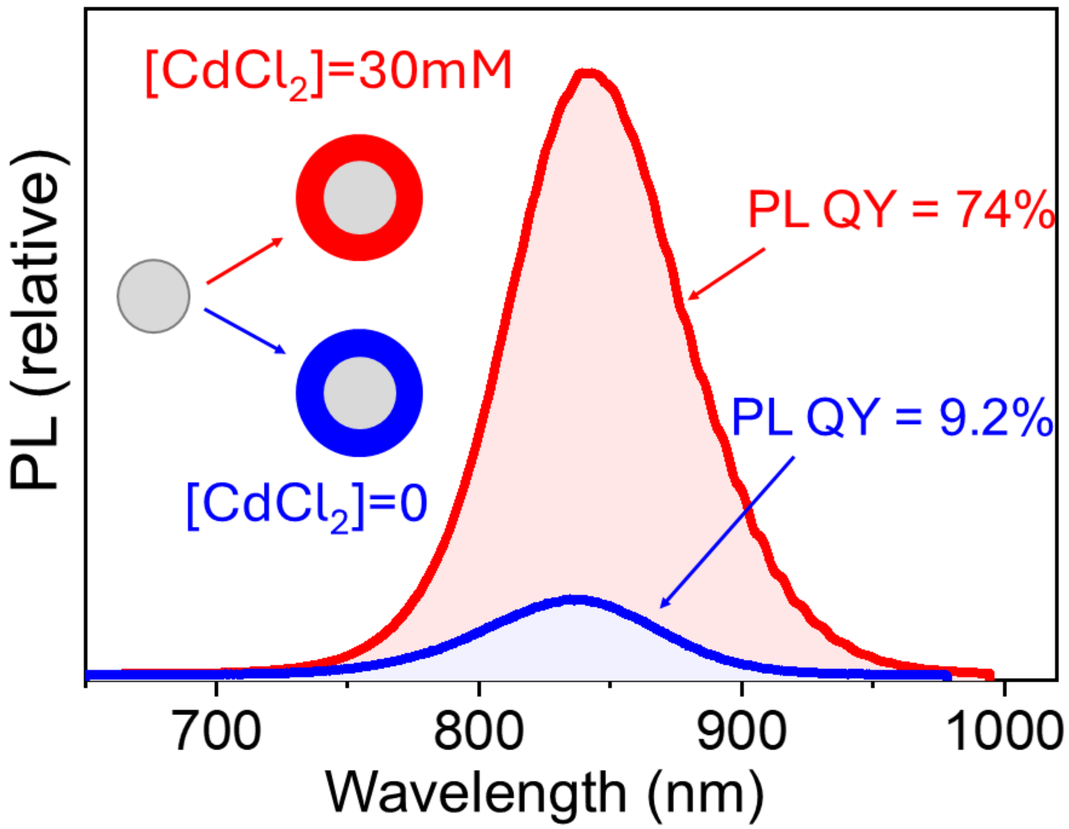

Figure S10. Comparison of the PL intensity and QY between $CdTe_{78}Se_{22}/Zn_yCd_{1-y}Se$ core/shell QDs grown by a standard hot-injection method (blue) and those grown in the presence of $CdCl_2$ (red). The halide-assisted route increases PL QY from ~ 9.2% to ~ 74%.

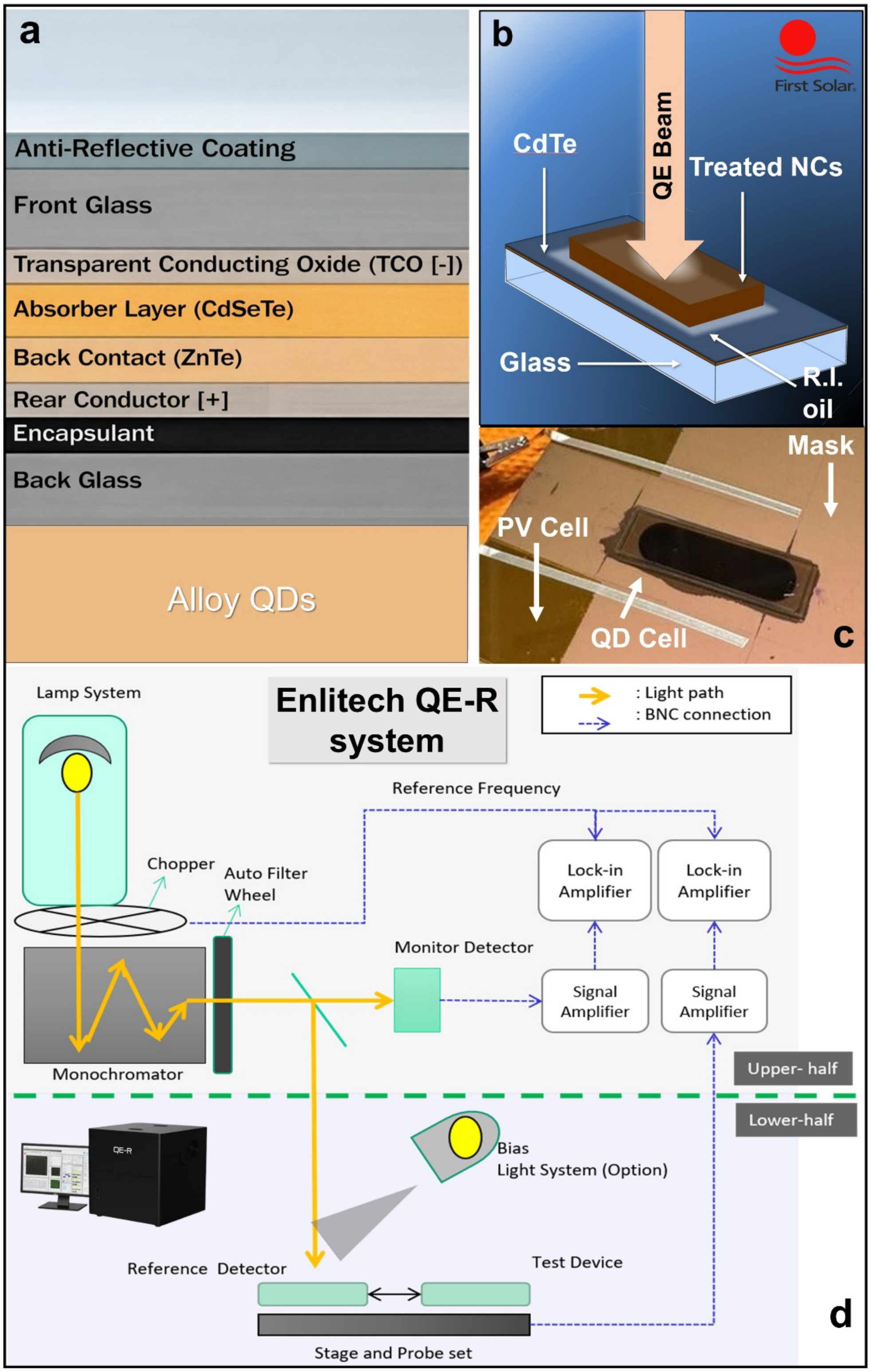

Figure S11. Cross-sectional schematic of a bifacial CdTe PV module. (*Solar Energy Materials and Solar Cells* **2023** *255*, 112289). (b) Schematic of the experimental setup to acquire film side quantum efficiency (FS-EQ) using a QD solution. (c) Photograph of the device. Image courtesy of First Solar. (d) Enlitech QE-R measurements system design.

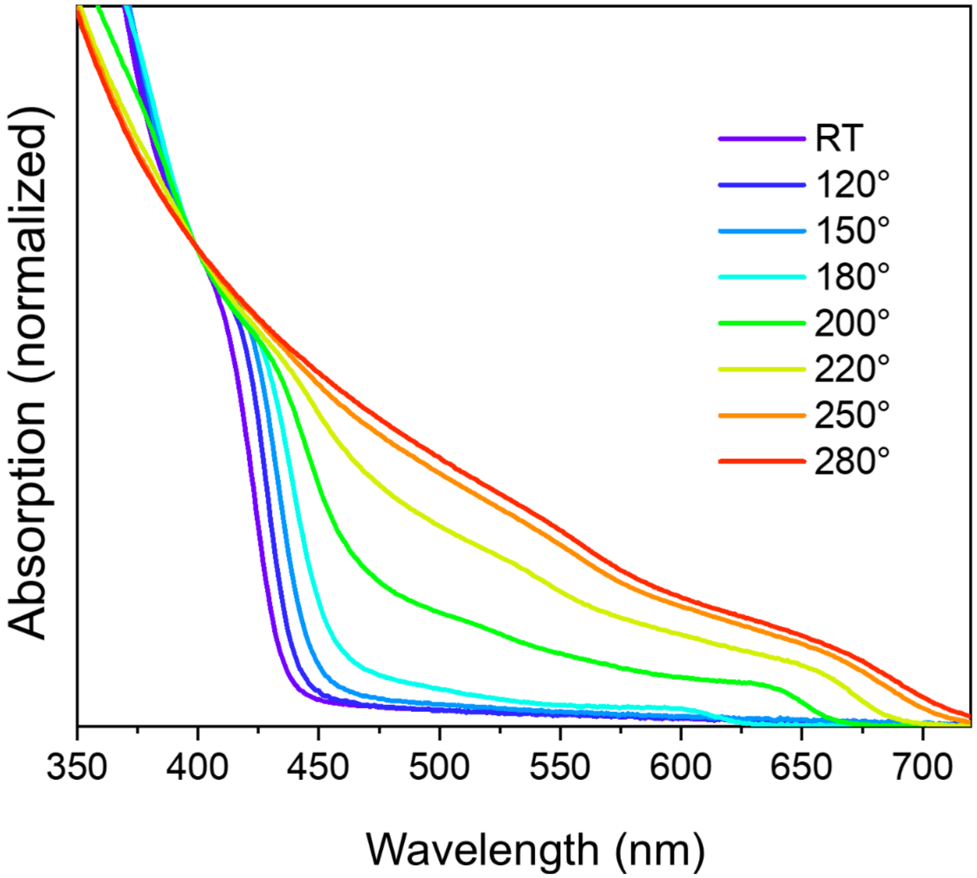

Figure S12. Temperature-dependent absorption evolution of ZnSe colloidal NCs under $CdCl_2$-assisted heat-up treatment. Absorption spectra are shown as a color-graded series, where cool colors correspond to the earliest spectra and warm colors to the latest spectra in each panel. The reaction leads to Zn→Cd cation exchange.

Relative and absolute efficiency gains.

Since the open-circuit voltage and fill factor do not change appreciably under backside illumination, the relative efficiency gain can be estimated directly from the measured raw increase in the back short-circuit current, assuming that approximately 20% of the incident photon flux reaches the back of the device.

$$\frac{\Delta\eta}{\eta} \approx \frac{\Delta J_{SC}}{J_{SC,0}} = \frac{f_{back} \times \Delta J_{back,raw}}{J_{SC,0}} = \frac{0.2 \times (9.0-3.3) mA/cm^2}{28\, mA/cm^2} \approx 4.1\% \qquad \text{Eq. S1}$$

This corresponds to 0.8-1.2% of the total efficiency gain, depending on the type of surface ground.

Section. I. The formation of molten phases in spherical NCs. From a thermodynamic standpoint, melting of inorganic nanocrystals can be understood in terms of interfacial energy minimization between the solid particle and its solvent environment. As illustrated in Fig S13a, melting occurs when the free-energy change, $\Delta G < 0$, is achieved through a reduction in the solid–solvent interfacial tension (γ_{SV}) by introducing a molten surface layer, δ, such that $\gamma_{SL} + \gamma_{LV} - \gamma_{SV} < 0$, where γ_{SL} and γ_{LV} are the solid-liquid and liquid-solvent tension terms. To estimate how the free energy varies with the thickness of the molten surface layer, δ, one can adapt the Kofman approximation[1] for spherical nanocrystals[2] in solution where $\Delta G(\delta) = Gslv\text{-}Gsv$ is examined as a function of δ. A typical evolution of ΔG with δ is illustrated in Figure S13b.[3] The local minimum of $\Delta G(\delta, T)$ provides the expected thickness of the molten layer, $\delta(T)$. If $\delta = R$, the entire nanoparticle is considered to be in a liquid phase.

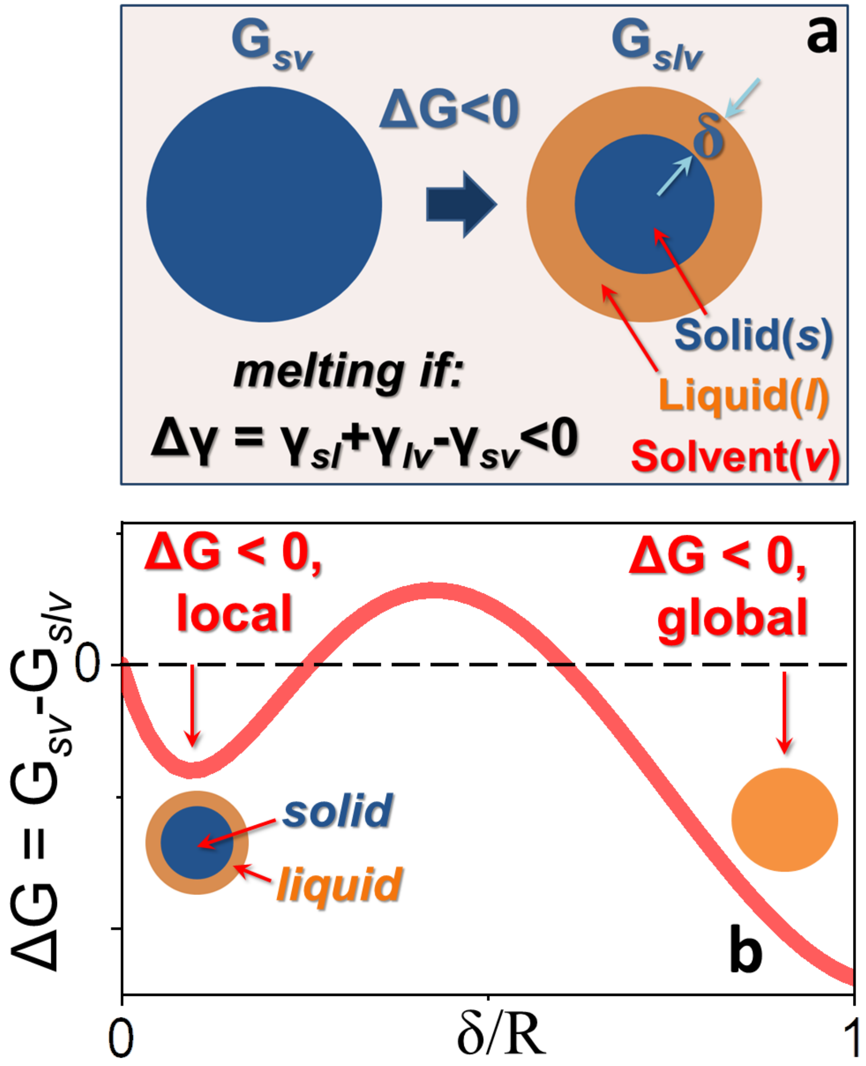

Figure S13. A diagram explaining the formation of molten phases in spherical NCs. (a). Graphical representation of the reduction in the nanoparticle Gibbs free energy (ΔG), which drives the formation of the molten layer with a thickness δ. Melting is observed if the solid/solvent interfacial tension (γ_{SV}) is reduced upon the insertion of a liquid layer: $\gamma_{SL} + \gamma_{LV} - \gamma_{SV} < 0$, where γ_{SL} and γ_{LV} are the solid/liquid and liquid/solvent interfacial tensions, respectively. b). Illustration of the local and global minima of ΔG that corresponds to melting of a surface layer (local minimum) and the entire particle (global minimum)

EXPERIMENTAL METHODS

Materials. The following chemicals were used as received without further purification or modification: anhydrous acetone (99 %, Amresco), cadmium oxide (CdO, 99.95%, MilliporeSigma), zinc acetate dihydrate (98%, Acros Organics), zinc chloride ($ZnCl_2$, ≥98%, MilliporeSigma), anhydrous ethanol (EtOH, 99%, BeanTown Chemical), hexane (ACS grade,

Thermo Scientific), 1-octadecene (ODE, technical grade, 90%, MilliporeSigma), octane (98%, MilliporeSigma), 1-octanethiol (97%, Alfa Aesar), oleic acid (OA, technical grade, 90%, MilliporeSigma), oleylamine (OLAM, technical grade, 70%, MilliporeSigma), dioctylamine (DOA, 97%, MilliporeSigma), rhodamine 101 inner salt (R101, 94%, Thermo Scientific), selenium powder (Se, 99.5%, 200 mesh, Thermo Scientific), sulfur powder (S, 99.999%, Thermo Scientific), toluene (99.8%, MilliporeSigma), and tri-n-octylphosphine (TOP, 97%, Strem Chemical). Tellurium powder (Te, 99.8%, MilliporeSigma), cadmium acetate ($Cd(OAc)_2$, ≥98%), and zinc acetate ($Zn(OAc)_2$, ≥98%) were also used as specified. High-purity argon was employed for inert-atmosphere operations.

CdS synthesis. A cadmium oleate-$Cd(OA)_2$ stock solution was prepared by loading 0.3 g of CdO, 5 mL of OA, 5 mL of ODE into a 50 mL 2 neck flask and heating it to 260°C under an Argon atmosphere. Added 10 mL of ODE once all the CdO has dissolved (indicated by a clear solution) and let the temperature recover back to 260°C. Meanwhile the S-ODE precursor was prepared in a separate 25 mL flask by measuring 0.080 g of Sulfur powder along with 5ml of ODE and heating up to 140°C till all the sulfur powder has dissolved and cooled down to 90°C in preparation for hot injection. Injected the S-ODE solution into the $Cd(OA)_2$ stock solution and cooked for a 2.5 minutes before quenching the reaction with a warm water bath. Seed CdS NCs were separated by centrifugation with EtOH and toluene and stored in either hexane or toluene.

CdTe seed synthesis. A Te-TOP stock was prepared in a 25 mL three-neck flask by mixing 0.64 g tellurium powder, 6 mL TOP, and 6 mL ODE and heated 265°C under Argon until dissolution (~30 min). The stock solution was then cooled down to 100°C and Argon atmosphere was maintained. At the same time, $Cd(OA)_2$ Precursor was prepared in a 100 mL three neck flask by combining 1.28 g CdO, 20 mL OA, and 30 mL ODE and degassing under vacuum at 120°C for 5 min. Switched to argon and heated to 260°C to completely dissolve all CdO, and cooled down to 100°C. For nucleation, the Te-TOP solution was swiftly injected into the hot $Cd(OA)_2$, and the temperature was raised to 200°C, and the flask was removed from heat and cooled in a warm water bath once the 200°C was reached. The CdTe NCs were separated from the crude solution by precipitation with EtOH and toluene at 6500 rpm and redissolved in 18 mL toluene.

CdSe seed synthesis. A Se–TOP stock was solution was prepared in a 25 mL three neck flask loaded with 0.59 g selenium powder, 4 mL TOP, and 3 mL ODE heated to 140°C under argon until complete dissolution (~20 min). Flask was then cooled down to 50°C and maintained under Argon. At the same time, a $Cd(OA)_2$ solution was prepared in a 50 mL three neck flask by combining 0.64 g CdO, 10 mL OA, and 15 mL ODE and heating under argon to 260°C until clear. For nucleation, the Se–TOP stock solution was quickly injected into the hot $Cd(OA)_2$, allowed to react for 10 s, then removed from heat and cooled with a warm water bath. The CdSe NCs were precipitated by centrifugation with EtOH and toluene and redissolved in 12.5 mL toluene.

CdTeSe alloy NCs via seed coalescence. A fusion medium was prepared in a 50 mL three neck flask by combining 125 mg $ZnCl_2$, 15 mL OLAM, and 3 mL TOP and heating under argon to 310°C. Separately, 2.26 mL of CdTe seed dispersion and 0.436 mL of CdSe seed dispersion were each precipitated with EtOH by centrifugation, combined, and redissolved together in 3 mL ODE. Upon reaching 310°C, the seed solution mixture was injected into the fusion medium, the temperature drop to ~260°C was allowed to recover back to 310°C, and aliquots were monitored

by absorption/PL. When the first exciton emission peak reached ~810 nm, heating was stopped, and the mixture was cooled to 200°C, and the reaction was quenched by a warm water bath. The crude product was transferred to a 15 mL centrifuge tube, 1 mL toluene was added, and the dispersion was precipitated with ethanol at 1:1 (v/v), by centrifuging for 5 min. The supernatant was discarded, and the QD pellet was redispersed in toluene or hexane. this cleaning step was repeated 1–2 times.

CdTeSe/CdZnSe growth (alloy shell growth). For the deposition of $Cd_yZn_{1-y}Se$ protective shell, the crude CdTeSe reaction mixture was transferred to a 250 mL flask. A sonicated solution containing 335 mg $Cd(OAc)_2$, 0–600 mg $Zn(OAc)_2$, 10 mL ODE, 10 mL OLAM, and 12 ml of OA was added. The mixture was pumped at 120°C until bubbling ceased and then placed under argon, heated to 235°C, and a 0.03 M Se–TOP precursor was injected dropwise at 12 mL/h for a total of 1 h. Injection was stopped, the flask was removed from heat and let cool down. Particles were precipitated with EtOH and toluene, and the product was redissolved in toluene.

The effect of various halide salts on the particle size evolution (Figure 2). (c) 20 mg of MX (M = Zn, Cd, or In; X = F, Cl, or I) was loaded into a 25 mL three neck flask along with 3 mL of OLAM and 0.6 mL of TOP. The mixture was placed under Argon atmosphere. The reaction mixture was heated to 260°C until the metal halide was fully dissolved, after which the flask was cooled down to room temperature. Separately, 660 nmol of CdSe was dissolved in 1 mL of ODE. This precursor seed solution was injected into the cooled reaction mixture, and the temperature was subsequently raised back to 260°C. Aliquots were withdrawn at 20°C intervals. The control test without halides present is shown in yellow.

(d) 20 mg of $CdCl_2$, 3 mL of OLAM, and 0.6 mL of TOP were loaded into a 25 mL three neck flask. The reaction mixture was heated to 260°C, after which 900 nmol of CdSe seed NCs dissolved in 1 mL of ODE were swiftly injected. The solution was maintained for 10 min, and aliquots were collected every 50 seconds. Photoluminescence (PL) spectra were recorded for each aliquot, and the full width at half maximum (FWHM) values. An identical experiment was performed in the absence of $CdCl_2$ to serve as a control.

(e) 20 mg of $CdCl_2$, 3 mL of OLAM, and 0.6 mL of TOP were loaded into a 25 mL three neck flask. The mixture was heated to 260°C and then cooled to 100°C. At this point, 900 nmol of CdSe seed NCs dissolved in 1 mL of ODE were injected. The reaction mixture was subsequently heated to 280°C.

CdSe/CdS giant Core/Shell QD (Figure 4e). 80 nmols of CdSe seed NCs were loaded into 50 mL 2 neck flask along with 2 mL of ODE and 2ml of OLAM and heated to 315°C under Argon. Once the reaction temperature has reached 300°C, started injecting 0.1 M $Cd(OA)_2$ precusor and 0.12 M Octanethiol-ODE solution separately with the use of a dual syringe pump at a rate of 3 mL/hr. Injections was continued for over 4 hours to obtain thick CdS shelling over the CdSe starting material. Injection was stopped and temperature was maintained at 315°C for 40 mins removed from the heating mantel and injected 2 mL of OA at 250°C cooling down. Products were separated from the crude solution via precipitation with EtOH and Toluene and stored in toluene or hexane for further use.

Integrated CdTe PV module QE measurements: CdSeTe NCs were dispersed in hexane and loaded into a 500 μm path-length circular dichroism quartz cuvette from FireFlySci for UV–Vis measurements and Film-Side external Quantum Efficiency (FS-QE). UV–Vis spectra were collected using a center-mounted cuvette holder on a PerkinElmer Lambda 1050+ spectrophotometer equipped with a 150 mm integrating sphere and dual detectors: an R6872 photomultiplier tube (PMT) and a Peltier-cooled InGaAs detector. FS-QE was measured using an Enlitech QE-R system referenced to a silicon standard cell, without light bias, at 25°C (Figure S11d). FS-Jsc is calculated by integrating the FS-QE spectrum with the AM1.5G solar photon flux. When measuring the FS-QE with a quartz cuvette, the cuvette was placed onto the back of the device with a small amount of refractive index matching oil (Cargille R. I. Series A (n=1.56)).

References.

[1] Weitz, D. A.; Huang, J. S.; Lin, M. Y.; Sung, J. Limits of the Fractal Dimension for Irreversible Kinetic Aggregation of Gold Colloids. *Phys. Rev. Lett*. 1985, 54, 1416–1419.
[2]Buffat, Ph.; Borel, J.-P. Size Effect on the Melting Temperature of Gold Particles. *Phys. Rev. A* 1976, 13, 2287–2298.
[3] Cassidy, J.; Harankahage, D.; Ojile, J.; Porotnikov, D.; Walker, L.; Montemurri, M.; Narvaez, B. S. L.; Khon, D.; Forbes, M. D. E.; Zamkov M. "Shape Control of Colloidal Semiconductor Nanocrystals through Thermodynamically Driven Aggregative Growth." *Chem. Mater*., 2022, 34, 2484-2494.
